# Human Motion Tracking with Less Constraint of Initial Posture from a Single RGB-D Sensor

**DOI:** 10.3390/s21093029

**Published:** 2021-04-26

**Authors:** Chen Liu, Anna Wang, Chunguang Bu, Wenhui Wang, Haijing Sun

**Affiliations:** 1 College of Information Science and Engineering, Northeastern University, Shenyang 110819, China; liuchen_sia@foxmail.com (C.L.); angleboy@foxmail.com (W.W.); seamirror@126.com (H.S.); 2 State Key Laboratory of Robotics, Shenyang Institute of Automation, Chinese Academy of Sciences, Shenyang 110016, China; 3 Institutes for Robotics and Intelligent Manufacturing, Chinese Academy of Sciences, Shenyang 110169, China

**Keywords:** 4D reconstruction, human motion capture, RGB-D sensor

## Abstract

High-quality and complete human motion 4D reconstruction is of great significance for immersive VR and even human operation. However, it has inevitable self-scanning constraints, and tracking under monocular settings also has strict restrictions. In this paper, we propose a human motion capture system combined with human priors and performance capture that only uses a single RGB-D sensor. To break the self-scanning constraint, we generated a complete mesh only using the front view input to initialize the geometric capture. In order to construct a correct warping field, most previous methods initialize their systems in a strict way. To maintain high fidelity while increasing the easiness of the system, we updated the model while capturing motion. Additionally, we blended in human priors in order to improve the reliability of model warping. Extensive experiments demonstrated that our method can be used more comfortably while maintaining credible geometric warping and remaining free of self-scanning constraints.

## 1. Introduction

Since we live in three-dimensional space, 3D representations of real-world objects are more comfortable for humans to understand. This leads to the challenging task of 3D scanning and modeling widely used in the fields of 3D printing, measurement, and games. In recent years, low-cost consumer-level RGB-D sensors that combine easiness and portability have made it easier to represent objects in three dimensions. Kinect Fusion [1,2] are pioneers in consumer-level sensor reconstruction static scenes. However, the scene or subject may move or deform in a non-rigid way. The limitations of static or rigid scenes hindered the widespread application of reconstruction. Follow-up research [3,4,5,6,7] extended reconstruction to non-rigid scenes. In real life, the common and essential non-rigid object is the human body. Due to the diversity and complexity of the human body, especially in practical applications where it is impossible to carry out too strict initialization, it is not easy to completely reconstruct a non-rigid human body. Researchers have devoted great effort in attempting to overcome these challenges. Some researchers published their data, attracting more people to engage in related research [8].

Early research [9,10,11] relied on a pre-scan template to obtain a complete model, which put forward higher initialization requirements for users and made the method difficult to use. The recent volume measurement method eliminated the dependence on the pre-scan model by using an RGB-D sensor and high-performance GPU to capture the human body and maintain high efficiency. Recently, researchers have made full use of the advantages of RGB-D sensors through multi-view settings [6,12,13,14,15,16], but the high fidelity of the model requires a strict working environment. However, the high cost and difficulty of deployment has significantly restricted its application. This makes the method of reconstruction using only a single RGB-D sensor more attractive.

Many approaches [3,4,5,7,17,18,19,20] focus on the easiness of the method and employ a single RGB-D sensor setup to achieve a complete reconstruction with a temporal fusion pipeline. However, these single-view methods suffer from carefully turning around to obtain a complete reconstruction. When the capture is incomplete, it is difficult to accurately track the non-rigid deformation in new fused areas. Moreover, significant progress has been made in learning-based methods for predicting human attributes. This overcomes the limitation of not capturing the entire human body from a single perspective. These data-driven methods already encode prior information about the human body, such as posture and body shape. However, the methods based on only RGB input [21,22,23,24,25,26,27,28,29] did not achieve a reliable body shape estimation due to the ambiguity of scale. For another reason, the methods that employed depth input [30,31] have also made some progress, but the recovery of details is unsatisfactory. Many researchers turned their attention to the estimation of parameterized models due to the easiness and fast calculation of templates. The scale ambiguity caused by the inherent characteristics of RGB images is the main difficulty in body shape estimation [32,33,34,35,36,37,38,39,40,41]. However, the estimation of the body shape using the 3D input method can be trusted. Still, due to the limitation of the template, the body shape cannot be obtained in high fidelity.

In this paper, we propose a method using a single RGB-D sensor to capture a complete human surface with less constraint of initial posture. Our new pipeline overcomes the constraints of self-scanning while robustly capturing human movements under monocular settings. To make the system achieve a reliable tracking consequence, we integrated the implicit occupancy representation, human pose, and shape into our pipeline to achieve superior surface reconstruction and motion tracking. We used a data-driven implicit occupancy representation method to generate a complete human mesh with geometric details, thus initializing the motion capture parameters and human priors. Subsequently, we tracked the human body motion and combined the priors and depth to refine the geometric details of the model. To summarize, we make the following technical contributions:

- We propose a human volumetric capture method based on human priors, which can effectively reduce the strict requirements of the initial posture and keep accurate motion tracking.
- To overcome the self-scan constraint, we propose a new optimization pipeline that combines human priors with volume fusion only using the front-view input.

## 2. Related Research

We can roughly divide similar efforts into three categories: data-accumulation-based approaches, learning-based approaches, and template-based approaches.

### 2.1. Data Accumulation Based Approaches

The method based on data-accumulation can undoubtedly capture the details of a scene effectively [1,2] and with the huge number of vertices of the model. It is not accessible to track the user’s motion in a timely manner. Due to the ambiguity of the data and so on, improving efficiency by reducing the dimensionality of the model may lead to natural deformation. Researchers have made various efforts in pursuit of high efficiency and accurate deformation. For example, Li et al. [42] allowed ordinary users to capture their complete and fully textured 3D models and to maintain models that were robust to minor deformations and attitude changes. Zhang et al. [43] pre-scanned the human body and registered the preliminary scan to get the watertight model. They used the model to train a SCAPE-like parameterized model and achieved a dynamic effect by changing the model parameters. Dou et al. [4] used bundle adjustment to optimize the non-rigid deformation parameters and to allow a considerable amount of non-rigid deformation during scanning. Newcombe et al. [1] reconstructed scene geometry and estimated a dense volumetric 6D motion field that warped the captured geometry into a live frame. Guo et al. [44] adopted a $l_{0}$ norm constraint to generate articulate motions without embedded skeleton. Innmann et al. [5] parameterized the geometry and motion by uniformly encoding the distance field as well as non-rigid deformation and then combined sparse color features and dense depth to track the human motion. Dou et al. [6] initialized non-rigid alignment by estimating the dense corresponding field of each individual RGB-D frame through a learning-based technique, allowing fast motion. Slavcheva et al. [7] solved the non-rigid registration problem by estimating the dense deformation field aligned with a pair of shapes of interest. Slavcheva et al. [45] twisted the given TSDF to the target TSDF through the Sobolev gradient flow so that it could handle any geometry, including topological changes. Using a template to constrain non-rigid deformation is a good idea, but it also requires a strict initialization posture to obtain an effective deformation field [19]. The self-scanning constraint is an inherent defect of this type of method. The data-driven method is used for initialization to solve this problem [46], but it uses the same method as DoubleFusion to compute non-rigid deformation of the model and has the same problem in terms of initializing attitude.

### 2.2. Learning-Based Approaches

The learning-based methods have developed rapidly in recent years, considering their recognition accuracy and operation efficiency, and have achieved good outcomes. Most methods use RGB images or depth images as input to learning an occupancy volume. Varol et al. [20] are pioneers in this research. They proposed a neural network that uses a single color-image to predict volume body shape. Zheng et al. [22] generated discrete volumetric representations with increased resolution and details and restored details with surface normals. Saito et al. [21] developed a multi-level architecture to use an extensive background and used high-resolution input to ensure precise predictions; Zheng et al. [25] integrated the semantic features of the parameterized model into the network to improve the generalization ability in challenging poses and various clothing topological scenarios. Moreover, with imperfect body reference, detail reconstruction is enabled by incorporating the deep ambiguous perception training loss. Wang et al. [29] introduced an adversarial learning framework based on normal maps, which not only improves the front view depth de-noising performance but also infers back view depth images with impressive geometric details. Onizuka et al. [26] combined a CNN (convolutional neural networks) and PCN (corresponding part connection network) to learn a distribution of the TSDF in the tetrahedral volume from a single image. Huang et al. [27] used parametric 3D human body estimation to construct the semantic space and semantic deformation field, which allows the 2D/3D human body to be converted into a canonical space to reduce geometric blur caused by occlusion in pose changes. Chibane et al. [30] used the 3D multi-scale tensor of deep features for encoding and classified deep features extracted at their location. Moreover, it provided continuous output, which can handle various topological structures and can retain details. This type of method overcomes the self-scanning constraint, but the obtained human body mesh does not necessarily have a distinguishable human body structure, limiting its more comprehensive application. At present, the method that uses a single RGB image as the input is the mainstream, and the ambiguity of the scale of RGB images is an unavoidable limitation. Moreover, using only RGB images to restore the geometric details of the model does not seem to be a reliable method [21,22,23,24,26,27,28].

### 2.3. Template-Based Approaches

Generally, the parametric human body template has the advantages of easy use and fast calculation. Even though it cannot completely and accurately reconstruct the details of the human body, it can represent the body shape and posture to a certain extent. Hence, much human body reconstruction work has turned to estimate the parameters of the parametric model. Bogo et al. [32] used a color image to predict the position of 2D joint points and then fitted the SMPL model to the 2D pose. Bogo et al. [47] leveraged a multi-resolution parametric body model to enable the estimation of body shape and pose in a coarse-to-fine manner; Joo et al. [15] established the human body model “Adam” and constrained the model to the current pose with key-points, 3D point clouds, human body priors, and stitching restrictions. Pavlakos et al. [41] added face and hand key-points to the SMPL model, proposed the SMPL-X model, and trained the posture priors using a variational autoencoder. Kanazawa et al. [33] proposed an end-to-end framework from a single RGB image to a complete 3D human body mesh. Zhu et al. [37] predicted the corresponding depth image based on the RGB image and deformed the 3D mesh with depth to refine the model. Kolotouros et al. [38] used the predicted SMPL parameters to initialize before optimization, thus making the fitting faster and more accurate. Pavlakos et al. [34] paid attention to the consistency of the human body texture under different perspectives and proposed a new optimization loss idea. Omran et al. [39] proposed a neural network that integrated bottom-up semantic segmentation and top-down human model constraints to predict human 3D pose and body shape. Yoshiyasu et al. [40] used the dense correspondence between image points and human body surface to learn 3D human body poses from a 2D image. In order to use different network structures to extract better features for each regression task, Sun et al. [48] decomposed the regression of pose and body shape into two self-networks and consequently proposed a two-way contour constraint to limit the estimated body geometry. Kocabas et al. [35] proposed an adversarial learning framework and defined a new type of temporal network structure with a self-attention mechanism without ground truth labels. Choi et al. [36] first used a graph convolutional network (GraphCNN)-based system to estimate a 3D pose from a 2D one and solved the problem of 2D pose representation. The proposed system avoided the representation issues while fully exploiting the mesh topology with GraphCNN in a coarse-to-fine manner. The lack of details is an inherent defect of this type of method. The deformation of the learning template to the details makes up for this defect to a certain extent [24], but the estimation of the geometry of the details through a single RGB input does not seem to be reliable. What is more, the mainstream method is still dominated by a single RGB image, and the limitation brought by the scale ambiguity still cannot be ignored.

In general, the above three types of methods have their advantages and limitations. To stand upon the shoulders of giants, we blended the above three methods to overcome existing limitations. We used learning-based methods to overcome self-scanning constraints and employed human body templates to constrain the non-rigid deformation of the mesh to improve the efficiency of the algorithm, and finally refined the details of the complete mesh based on the method of data-accumulation.

## 3. Method

**Figure 1:**
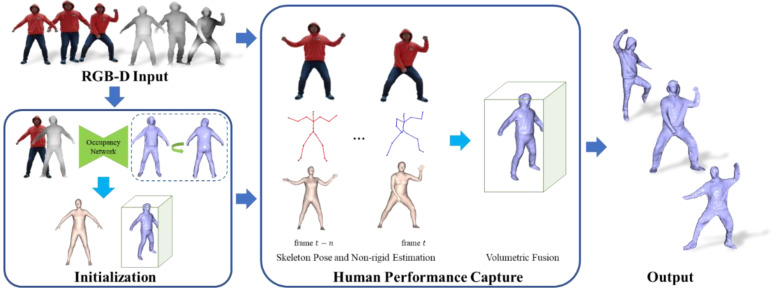
The pipeline of our method.

We used a single RGB-D camera as the only sensor and took the captured RGB-D image (a pair of RGB image and depth image D) as input to reconstruct a complete human body detail surface. The initial pose of the human has a significant impact on reconstruction. To increase the easiness of the system while maintaining high fidelity, we propose a novel reconstruction method combined with human priors. As illustrated in Figure 1, we first used the predicted complete human mesh and captured depth to initialize the TSDF volume (including the volume pose). Then, we captured the human performance, tracked the motion, and updated the model surface by fusing the newly observed depth.

### 3.1. Initialization

#### 3.1.1. Volume Alignment

To obtain a complete human mesh in the front view, we employed the NormalGAN [30] to generate a complete mesh with specific details. Because the complete mesh could not recover all the details yet, we initialized the TSDF volume by voxelizing the mesh to make the surface updated by realistic depth. We defined the voxel size as 0.005 m and determined the volume dimension according to the body size of the complete model. We used a nonlinear optimization method to align the generated complete mesh with the current depth, which initialized the volume pose. The energy function is as follows:

(1) $$E_{sdf-align}=chamfer\left(P_{data},Vis\left({\tilde{P}}_{comp}\right)\right)$$

(2) $$chamfer\left(P,Q\right)={\left|P\right|}^{-1}\underset{\left(p,q\right)\in \Lambda_{P,Q}}{\sum }\left\|p-q^{2}\right\|+{\left|Q\right|}_{\left(q:p\right)\in \Lambda_{P,Q}}^{-1}\left\|q-p^{2}\right\|$$

(3) $${\tilde{P}}_{comp}=SE\left(dq_{vol}\right)P_{comp}$$

where $P_{comp}$ is the vertex of the complete mesh obtained by the Marching Cube algorithm [49] from TSDF volume; $dq_{vol}$ is the dual quaternion of the volume from the original pose; SE(·) maps a dual quaternion to SE(3) space; $P_{data}$ is the point cloud of the human body that generated from depth; Vis(·) selects the points that are visible in the front view; $\Lambda_{P,Q}=\left\{\left(p,arg\;min_{q}\left\|p-q\right\|\right):p\in P\right\}$ is the set of pairs (p,q) where q∈Q is the nearest neighbor of p∈P.

#### 3.1.2. Inner Body Alignment

In particular, since we only reconstructed a single human object, using a human prior can constrain the unnatural mesh deformation effectively. Therefore, we adopted a two-layer motion representation of a hybrid ED model and SMPL [19]. For any vertex $v_{c}$ from a surface, let ${\tilde{v}}_{c}=ED\left(v_{c};W\right)$ denote the position after warping, where W is the non-rigid motion field. SMPL [50] is a parameter template that the body model $\bar{T}$ contains anthropometric laws. For any vertex $\bar{v}\in \bar{T}$ only depends on the shape parameters and pose parameters. To manipulate the template’s deformation, the shape parameter β and the pose parameter θ work together to adapt the model to different body shapes and poses, which denotes as $W\left(T\left(\bar{v};\beta ;\theta \right);\beta ,\theta \right)$.

It is crucial to align the SMPL with the complete mesh, which ensures the credibility of the complete mesh deformation. To make the fitting faster and more accurate, we used FrankMocap [51] to initialize the pose while reducing the amount of calculation. The energy function is as follows:

(4) $$E_{smpl-align}\left(\beta_{0},\theta_{0}\right)=\lambda_{data}E_{data}+\lambda_{joint}E_{joint}+\lambda_{shape}E_{shape}+\lambda_{prior}E_{prior}$$

Since the point cloud was used before to align the complete mesh, there was no need to use the same constraints again. The data item measures the alignment error between the complete mesh and the SMPL:

(5) $$E_{data}\left(\beta_{0},\theta_{0}\right)=chamfer\left({\tilde{P}}_{comp},W\left(T\left(\bar{v};\beta_{0};\theta_{0}\right);\beta_{0},\theta_{0}\right)\right)$$

where, ${\tilde{P}}_{comp}$ is the vertex of the aligned complete mesh.

The key-points of the human body not only hold the pose but also represent the shape of the human body to a certain extent. To estimate the body accurately, we penalized the distance between key-points and the corresponding joints of SMPL:

(6) $$E_{joint}\left(\beta_{0},\theta_{0}\right)=\underset{j\epsilon joint}{\sum }\gamma_{i}\omega_{i}\psi \left(R_{\theta_{0}}{\left(J\left(\beta_{0}\right)\right)}_{i}-J_{est,i}\right)$$

where ψ(·) denotes the robust Geman–McClure penalty function;$\;R_{\theta_{0}}{\left(J\left(\beta_{0}\right)\right)}_{i}$ is the 3D joints of SMPL for each joint i; $R_{\theta_{0}}\left(\cdot \right)$ is a function that transforms the joints along the kinematic tree according to the pose $\theta_{0}$; $J_{est}$ is the 3D joints that were obtained from Azure Kinect SDK. By the way, Azure Kinect SDK can track multiple people, provide corresponding point cloud masks, and simultaneously provide unique identification for each object. To reduce the adverse effects of noise, the contribution of each joint in the data term is weighted by the detection confidence score $\omega_{i}$, and $\gamma_{i}$ that are per-joint weights for optimization.

$E_{shape}\left(\beta_{0}\right)=\left\|\beta_{0}\right\|{}^{2}$ describes the Mahalanobis distance between the shape parameters being optimized and the shape distribution in the training dataset of SMPL.

Due to the ambiguity of the scale, the estimated body shape only using a color image cannot be fully believed, except for the pose. Therefore, we only penalize the attitude error between SMPL and FrankMocap and provide a prior for the attitude estimation:

(7) $$E_{prior}\left(\theta_{0}\right)=\underset{i}{\sum }\|\theta_{0}^{pose}-\theta_{frank}^{pose}{\|}_{2}^{2}$$

where, $\theta_{0}^{pose}$ and $\theta_{frank}^{pose}$ are the pose parameter of the inner body, and they are predicted pose by FrankMocap, respectively. Since the parameter θ of the SMPL includes the human pose, the global orient, and the model translation as a whole, we do not need to optimize all θ parameters. To make the optimization tractable, we used Pytorch and Pytorch3D [52,53], which can auto-differentiate and minimize the energy function $E_{smpl-align}$ to solve the initial body parameter $\beta_{0}$ and attitude parameter $\theta_{0}$ of SMPL.

**Figure 2:**
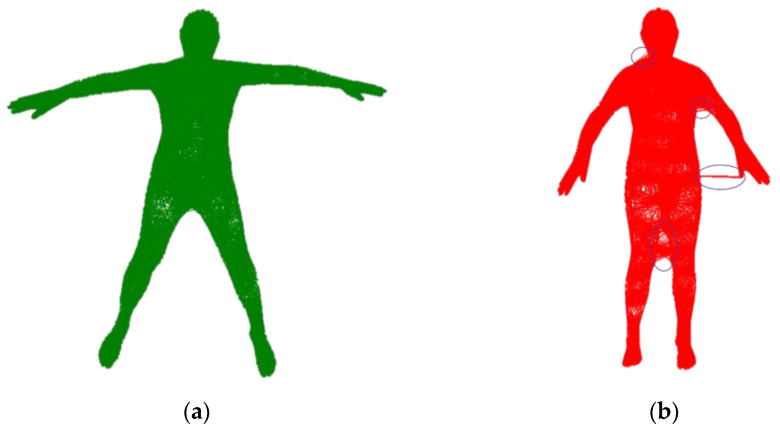
Vertex graph in different postures. (**a**) KNN nodes of vertex in a stretched pose. (**b**) Vertex graph constructed with fewer constraints of posture. The Figure shows that a loose posture may lead to unnatural KNN nodes of the vertex.

To qualitatively illustrate the necessity of the proposed method, we computed the KNN of the vertex $\hat{v}\in W\left(T\left(\bar{v};\beta ;\theta \right);\beta ,\theta \right)$ for different poses, as shown in Figure 2. To construct a correct KNN for the complete mesh, we pre-computed the KNN nodes with mesh vertex in a more stretched pose. Subsequently, we mapped the complete mesh to the inner body to construct a reliable KNN. To make the nodes distribute uniformly, we performed voxel grid downsampling on the inner body surface, which removes the high-frequency parts to obtain the sparse temporary node $\left\{{\hat{x}}_{i}\right\}$. To facilitate the tracking of nodes, we searched for the nearest vertex on the inner body surface to $\left\{{\hat{x}}_{i}\right\}$ as the final node $\left\{x_{i}\right\}$.

Along with occupancy volume, a semantic volume is initialized to hold semantics in the same dimension as TSDF volume. We searched the nearest inner body vertex of the voxel to find the corresponding and store it in semantic volume. This can enable the updated vertex to obtain a correct KNN conveniently.

Finally, to obtain a watertight human body mesh with geometric details, we used the method in RobustFusion [46] to blend a partial volume obtained from the current depth and TSDF volume. Thus, we obtained an updated mesh with details and use it for motion tracking in the next section.

### 3.2. Human Performance Capture

We propose a novel human performance capture scheme that robustly tracks human actions. Note that reliable initialization of human motion was provided in the previous section. In the motion tracking stage, the deformation is represented by the non-rigid deformation field generated by the nodes, and the deformation of the inner body is used as a strong constraint to control the warp of the mesh. After that, we use $v_{c}$ to denote any 3D point on the capture volume and ${\tilde{v}}_{c}$ to denote the position after using embedded deformation. For the skeletal motion, the skinning weights of $v_{c}$ are given by the weighted average of the skinning weights of its KNN nodes.

#### 3.2.1. Skeleton Pose Estimation

To obtain an accurate skeleton pose, we used point cloud and 3D pose to constrain θ and β, and employed the predicted human pose as a prior. The formula is as follows:

(8) $$E_{pose}=\lambda_{pdata}E_{pdata}+\lambda_{joints}E_{joints}+\lambda_{shape}E_{shape}+\lambda_{prior}E_{prior}$$

Here, the data item measures the misalignment error between the dense point cloud and the visible vertices of the inner body:

(9) $$E_{pdata}=chamfer\left(\pi^{-1}\left(D\right),Vis\left(W\left(T\left(\bar{v};\beta ;\theta \right);\beta ,\theta \right)\right)\right)$$

Here chamfer(·) is the same as Equation (2); Vis(·) selects the visible vertex of the inner body from the front view perspective; $\pi^{-1}\left(\cdot \right)$ converts the depth to a 3D point cloud. Point clouds are crucial for estimating skeleton pose, especially for body shape estimation and data alignment. $E_{joint}$, $E_{shape}$ and $E_{prior}$ are the same as Equation (4), restricting unnatural body shape and human pose.

#### 3.2.2. Non-Rigid Estimation

In order to capture the non-rigid deformation of reality, we solve the surface tracking energy as follows:

(10) $$E_{m}\left(W\right)=\lambda_{mdata}E_{mdata}+\lambda_{reg}E_{reg}+\lambda_{bind}E_{bind}$$

The data item measures the misalignment between the dense point cloud and the visible vertex of the non-rigid surface. For any 3D point $v_{c}$ in the capture volume, ${\tilde{v}}_{c}$ denotes the warped position after applying the ED motion field:

(11) $$E_{mdata}=chamfer\left(\pi^{-1}\left(D\right),Vis\left({\tilde{v}}_{c}\right)\right)$$

To prevent over-fitting to depth inputs, we borrow the energy terms from [47], which produces a locally as-rigid-as-possible motion:

(12) $$E_{reg}=\underset{e\in V^{\prime }}{\sum }\|{\left(AT\right)}_{e}-{\left(AN\right)}_{e}{\|}_{F}^{2}$$

where $V^{\prime }$ denotes the edges of the mesh constructed via node graph. AT and AN are the edge vectors of the triangles of $V^{\prime }$ in the origin pose (the pose that constructed node graph) and in the current pose, respectively. e indexes the edges.

To constrain coherent deformation, the binding term penalizes the error between the current node and the desired node:

(13) $$E_{bind}=\underset{x_{i}}{\sum }\|{\tilde{x}}_{i}-{\hat{x}}_{i}{\|}_{2}^{2}$$

where ${\hat{x}}_{i}$ is the desired node obtained from skeleton pose estimation, ${\tilde{x}}_{i}$ is the warped ED node by using non-rigid motion. All the pose and non-rigid optimizations in (4), (8), and (10) are solved using Adam on GPU.

#### 3.2.3. Volumetric Fusion

**Figure 3:**
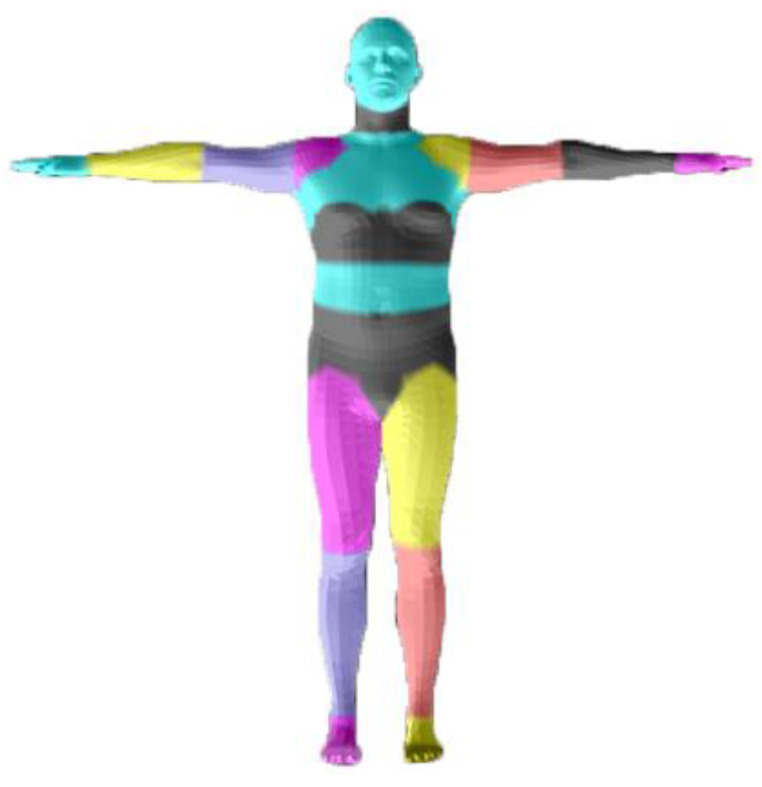
Human parsing label from inner body vertex segmentation.

To update the geometric details in time similar to RobustFusion [46], we fused the depth into TSDF volume and discarded the voxels that collided or warped invalid input to achieve a robust geometric update. We used a semantic-based motion tracking behavior method to avoid the deterioration of fusion caused by challenging motion. Different from RobustFusion, we directly used the depth to obtain human body parsing instead of depending on RGB image. This can avoid possible adverse effects due to camera calibration errors. We converted the depth to a point cloud and found the corresponding index for each point. Subsequently, we obtained the parsing label according to the segmentation of the inner body vertex in Figure 3. For each node $x_{i}$, $l_{i}$ is the corresponding label during initialization, and $L(\pi \left({\tilde{x}}_{i}\right)$ is the corresponding projection label of the current depth. For each voxel v, $\tilde{v}$ denotes its position that transformed by the warp filed. D(v) and W(v) denote the value of TSDF and accumulated weight; d(v) and w(v) denote the value of projective SDF and the updating weight. The updating formula can be expressed as:

(14) $$d\left(v\right)=\left(u-\tilde{v}\right)sgn\left(n_{u}^{T}\left(u-\tilde{v}\right)\right)$$

(15) $$w\left(v\right)=exp\left(\frac{-\|\Phi^{T}\left(\theta^{*}-\theta_{d}\right){\|}_{2}^{2}}{2\pi }\right)\underset{i\in N\left(v_{c}\right)}{\sum }\frac{\varphi \left(l_{i},L\left(\pi \left({\tilde{x}}_{i}\right)\right)\right)}{card\left(N\left(v_{c}\right)\right)}$$

(16) $$D\left(v\right)\leftarrow \frac{D\left(v\right)W\left(v\right)+d\left(v\right)w\left(v\right)}{W\left(v\right)+w\left(v\right)},W\left(v\right)\leftarrow W\left(v\right)+w\left(v\right).$$

where *u* is the corresponding 3D voxel of $\tilde{v}$ on the complete mesh, and $n_{u}$ denotes its normal; sgn(·) is a sign function to distinguish the positive and negative of SDF; $\theta^{*}$ is the optimized pose;$\;N\left(v_{c}\right)$ is the set of the KNN nodes of v;  φ(·) denotes an indicator, which equals to 1 only if the two input labels are the same. Finally, the voxel is updated by using a dynamic atlas scheme way. Please refer to [3,46] for more detail.

## 4. Experiment

In this section, we evaluate our system qualitatively and quantitatively. For the experiment, we used Azure Kinect as a single sensor and asked the user to perform specific actions in front of the sensor. Our experiments were executed on a PC with a NVIDIA GeForce GTX TITAN Xp GPU and an Intel Core i9-9900K CPU. In specific, the volume alignment took 0.7341 s, the inner body alignment took 1.2146 s, the skeleton pose estimation took 2.3441 s, and the non-rigid estimation cost 2.4732 s. The parameters used in the paper were set with $\lambda_{data}=5.0$, $\;\lambda_{joint}=8.0$, $\;\lambda_{shape}=10$, $\;\lambda_{prior}=5.0$, $\lambda_{pdata}=1.0$, $\;\lambda_{mdata}=1.0$, $\;\lambda_{reg}=10$, $\;\lambda_{bind}=1$.

### 4.1. Qualitative Evaluation

**Figure 4:**
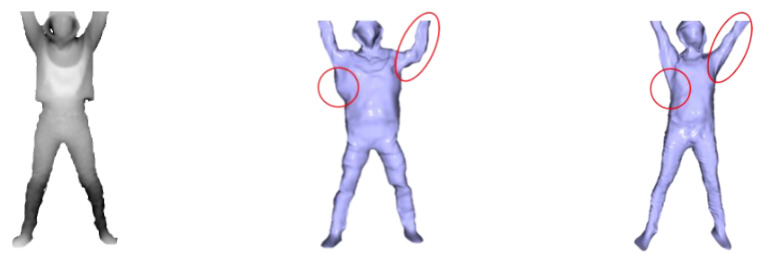

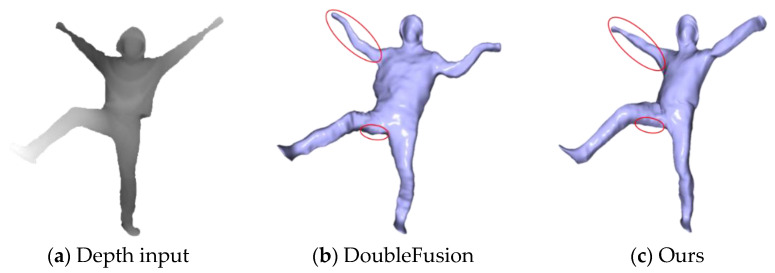
Qualitative evaluation.

We compared our system with the current state-of-the-art DoubleFusion [19]. To clearly compare the experimental effect, we asked the user to wear light clothes and then start capturing the depth sequence in the A-pose, but the arm was more similar to natural drooping. Then the user performed several joint movements. As demonstrated in Figure 4, our method has significant advantages in motion tracking results. This is mainly due to DoubleFusion having computed the KNN of the vertex by using the distance between the node and the ambiguous canonical vertex straightforward. As a result, some vertices obtained an incorrect warping that was affected by erroneous node transformation, leading to inaccurate tracking. To make our system more practical, we implemented a method that combined human semantics. This allowed the system to obtain a reliable KNN of vertex in volume surface with a freer posture.

**Figure 5:**
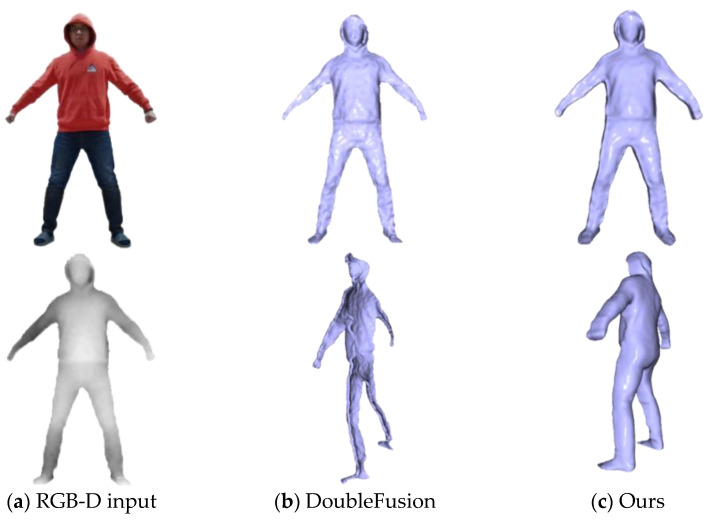
Evaluation of model completion.

**Figure 6:**
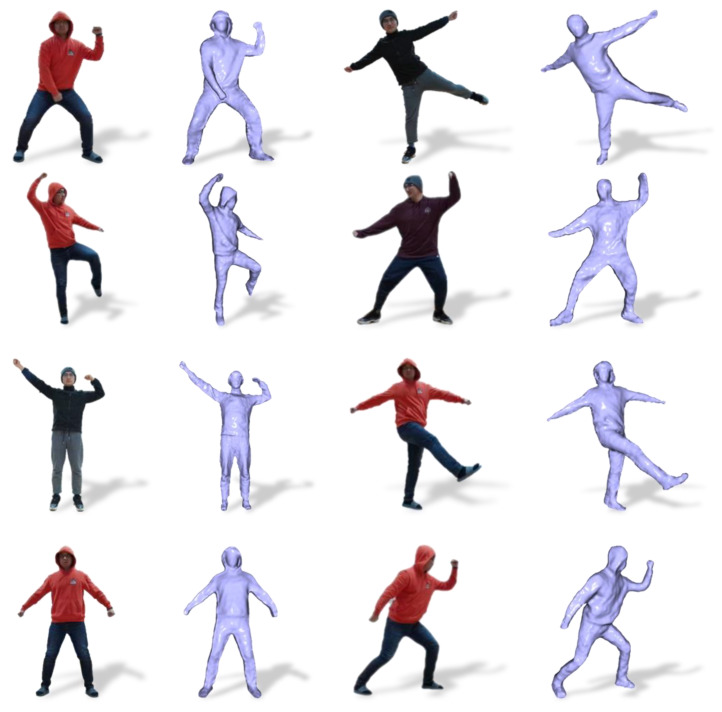
Fine-detailed geometry.

Moreover, DoubleFusion is a traditional reconstruction method based on data accumulation. This leads the system to have to rely on the perspective changing to fuse the visual depth gradually. Strict initialization assumptions also restrict the system from applying situations for which complete data of the user cannot be obtained, such as lying down. To achieve a watertight and fine-detailed human mesh at any time, we initialized the volume with the generated complete mesh. As can be seen from Figure 5, DoubleFusion captured visible data in a single view and obtained a partial geometry, while our method obtained a watertight mesh. In motion tracking, we updated the surface with the newly observed depth in different poses and obtained a fine-detailed geometry, as shown in Figure 6.

### 4.2. Quantitative Evaluation

**Figure 7:**
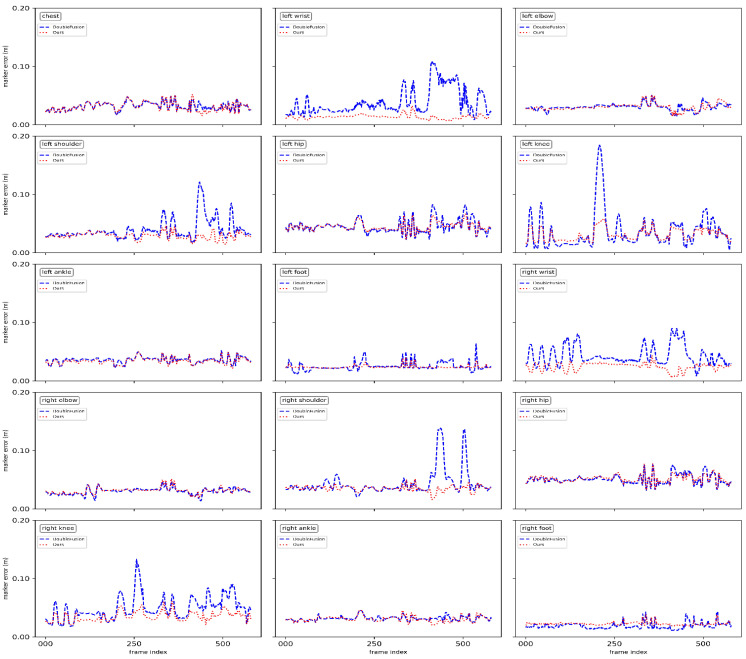
Curves of joints error.

To evaluate the accuracy of motion-tracking, we measured the errors between the human marker obtained with the VICON system and the model marker of the complete mesh. In the experiment, we asked the actor to perform body stretching, boxing, basketball, etc. As shown in Figure 7, both DoubleFusion and our method maintained a small error in the part where the correct deformation field was constructed; in contrast, the body parts with an incorrect warp field had larger errors. For a more detailed comparison, we list the maximum instant error of each joint of the entire sequence in Table 1. Affected by the error warp field, the contrast of the knee error is obvious. At the point where the correct deformation field was constructed, the maximum instant errors of the two methods were maintained at a similar level. Additionally, the maximum instant error of the wrist and the shoulder appeared almost simultaneously and had a clear contrast. The fast motion made the estimation of the skeleton pose unreliable, which led to the increase of the motion tracking error. The pose prior used by our method effectively prevented erroneous motion tracking.

**Figure 8:**
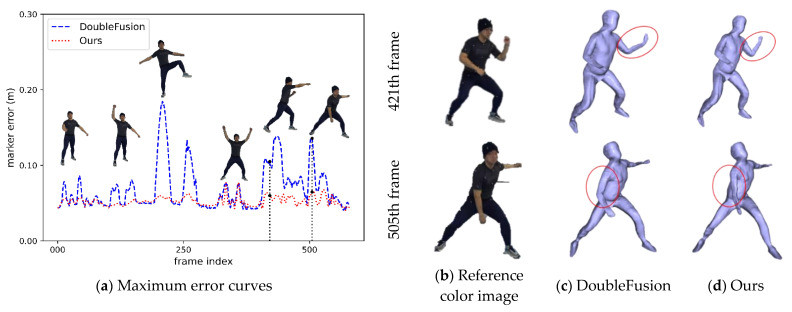
Results of motion tracking.

We also compared the maximum error of each frame, as shown in Figure 8. The maximum error occurred more in the knee and shoulder. This is because the incorrect warp field constructed by the bad initialization posture led to an incorrect deformation. The closer the two legs were, the higher the probability of establishing an incorrect warp field. The vertex belonging to the elbows and feet with a correct warp field deformed correctly under the constraints of the nodes and the point cloud, resulting in a minor error. The pose prior had a significant positive effect on motion tracking. We used confidence to balance joint constraints and human pose priors. When the confidence was low, we relied on the human pose prior to avoiding excessive tracking error.

For each frame, we calculated the maximum and the average errors of all the markers. We calculated averages for all the frames on the entire sequence. Table 2 lists the average maximum error and the average error of the sequence. We can see from the numerical error curves and the average errors that our system generated lower tracking errors compared with DoubleFusion.

## 5. Discussion

### 5.1. Limitations

One of the limitations of our system is that when the user is wearing very loose clothes (e.g., long skirt), it could lead to unnatural motion capture. This is mainly attributed to the ambiguity on the input depth. We believe this issue of unnatural motion capture can be solved by using separate clothes and limbs to establish a separate parsing. Specifically, the generated mesh may distinctly differ from the naked human body, which leads to unnatural human parsing. Additionally, our system cannot handle more detailed level surface motion capture, such as capturing the wrinkles in cloth. 

### 5.2. Conclusions

In this paper, we propose a motion capture and tracking system that combines human semantics with integrated depth to volume for human motion capture. We blended the learning-based method to overcome the self-scanning constraint of achieving a complete mesh (so that the system can achieve a watertight mesh at any time), which expands the application scenarios of the system. Moreover, we integrated semantics into the complete mesh, which can help the vertex of the mesh obtain a credible KNN, while increasing the easiness and robustness of the system. Finally, in the motion tracking stage, we did not depend on any input other than the depth to obtain the human parsing image. This can avoid undesirable fusion caused by calibration errors.

Additionally, we used a minor step in nonlinear optimization to pursue stability and a minor error in optimization. The result led to a longer running time of the software. To pursue higher efficiency, we plan to hunt for a better optimization approach in future work.

## Figures and Tables

**Table 1:** Maximum instant errors on the entire sequence of each joint.

Joint	DoubleFusion [2]	Ours
Chest	0.0495	0.0516
Left wrist	0.1078	0.0321
Left elbow	0.0507	0.0513
Left shoulder	0.1211	0.0459
Left hip	0.0822	0.0662
Left knee	0.1845	0.0582
Left ankle	0.0515	0.0492
Left foot	0.0633	0.0402
Right wrist	0.0896	0.0429
Right elbow	0.0471	0.0513
Right shoulder	0.1381	0.0514
Right hip	0.0755	0.0770
Right knee	0.1336	0.0604
Right ankle	0.0454	0.0457
Right foot	0.0423	0.0379

The measurement unit of the maximum instant error is expressed in meters.

**Table 2:** Average numerical errors on the entire sequence.

Method	DoubleFusion	Ours
Maximum Error (m)	0.0689	0.0526
Average Error (m)	0.0362	0.0316

## Data Availability

Not applicable.
